# Socioenvironmental aspects of the Purus Region - Brazilian Amazon: Why relate them to the occurrence of American Tegumentary Leishmaniasis?

**DOI:** 10.1371/journal.pone.0211785

**Published:** 2019-02-07

**Authors:** Jorge Augusto de Oliveira Guerra, Maria das Graças Vale Barbosa Guerra, Zanair Soares Vasconcelos, Nayra da Silva Freitas, Fernanda Rodrigues Fonseca, Rubens Celso Andrade da Silva Júnior, Arineia Soares da Silva, Vanderson Sampaio, Marcel Gonçalves Maciel, Melissa de Sousa Melo Cavalcante, Bernardino Cláudio de Albuquerque, Gilton Mendes dos Santos, Luiza Garnelo

**Affiliations:** 1 Postgraduate Program in Tropical Medicine, Universidade do Estado do Amazonas–UEA–Manaus–AM, Brazil; 2 Fundação de Medicina Tropical Dr. Vieira Dourado—FMT HVD,–Manaus–AM, Brazil; 3 Faculdade Metropolitana de Manaus–FAMETRO,–Manaus–AM, Brazil; 4 Hospital Militar de Manaus- HMAM,–Manaus–AM, Brazil; 5 Universidade Federal do Amazonas–UFAM,–Manaus–AM, Brazil; 6 Instituto Leônidas e Maria Deane/Fundação Oswaldo Cruz Manaus–ILMD/FIOCRUZ,–Manaus–AM, Brazil; 7 Fundação de Vigilância em Saúde—FVS-AM,–Manaus–AM, Brazil; University of Minnesota, UNITED STATES

## Abstract

This study aims to analyze factors related to the occurrence of American Tegumentary Leishmaniasis in the Purus Region, based on the reporting of cases between 2001 and 2013, correlating them with livelihoods and subsistence farming in the region, and analyzing them in regards to sex, age, clinical form, occupation, diagnostic methods and seasonality. The analysis parameter which was used included all cases of American Tegumentary Leishmaniasis in each sub-region by municipality. The Purus Region, between the states of Amazonas and Acre, consists of three sub-regions: Upper, Middle, and Lower Purus. We observed that socio-environmental impacts influenced the livelihoods of the human population and that the interaction with extractive activities, especially latex and Brazil nut collecting, where the labor regime implies a long stay in the jungle, leads to socio-environmental conditions that are favorable to the contraction of American Tegumentary Leishmaniasis. During the referred period, there were 13,971 cases of American Tegumentary Leishmaniasis distributed among the sub-regions: High (12611 cases = 90.27%), Middle (1225 cases = 8.77%) and Lower (135 cases = 0.96%). Among the 22 municipalities that were studied, Rio Branco stands out with 31.6% of cases, followed by Xapuri with 12.6% and Sena Madureira with 12.5%. In the results, we highlight the high percentage (20.8%) of mucous forms; the age group from 11 to 50 (70.2%), however, 20.2% were in the age group of 1 month to 10 years of age; cases in males were 69.8%, and, in the reports examined, 43.7%. cited their occupation as extractivism. A statistically significant negative association was demonstrated between cutaneous leishmaniasis and rainfall between Purus municipalities. However, in regards to the association of cutaneous leishmaniasis for both, temperature and Municipal Human Development Index—MHDI, no significant associations were found in Purus. We concluded that American Tegumentary Leishmaniasis occurring in the Purus Region is related to two distinct aspects: the development of enterprises that extend the agricultural frontier, and a change in lifestyle, namely the extraction of wood as an occupation, which has, as a consequence, an environmental impact and creates difficulties in accessing treatment.

## Introduction

American Tegumentary Leishmaniasis (ATL) is a zoonotic, vector-borne disease, caused by several species of *Leishmania* (Kinetoplastida: Trypanosomatidae) parasites, in the same geographical area. It is transmitted by sand flies (Diptera: Psychodidae), and is considered a neglected, infectious disease. The epidemiology is complex, with intra- and inter-specific variation in transmission cycles, clinical manifestations and response to therapy. The number of cases, incidence or density rates have a direct effect on the analysis of the leishmaniasis composite index in the regional context [1].

In Brazil, there are 7 species of *Leishmania* and in the Amazon region *Leishmania (Viannia)brasiliensis*, *L*. *(V*.*) guyanensis and L*. *(Leishmania) amazonensis* are the most important as acausal agents to ATL [2].

In the Northern Region of Brazil, ATL is associated with the expansion into previously healthy areas, maintaining high coefficients over the last years [3,4]. The urban, peri-urban and peridomiciliary transmissions persist, increasing the number of cases associated with widespread deforestation; occupancy inflows increased during the 1980s and 1990s, leading to the proliferation of certain species of vectors of the disease, as observed by other authors in different studies [5,6]. The opening of roads, the implantation of agricultural areas, military training and the installation of housing nuclei in forest areas are important factors related to the epidemiological profile of ATL in the Brazilian Amazon [3,7–9], since its transmission cycle occurs between vectors (phlebotomines) and wild animals (rodents). In this context, most of the time, people become infected when they either alter the natural environment, interpose the wildlife cycle and/or enter this other ecosystem [10,11].

For the western Amazon, few studies report information on the disease and no published data covers the entire Purus region [9]. Previous studies related work activities, such as extraction of natural resources, to 46 patients mostly from Lábrea, with the mucosal form of the disease. These 46 cases presented strains of *Leishmania (Viannia) braziliensis* and *Leishmania (Viannia) guyanensis* which were obtained from biopsies of the lesions [12,13]. Another study involving 234 patients with mucosal leishmaniasis showed that activities involving agriculture, hunting, or mineral extraction can put residents or workers at risk of contracting American Tegumentary Leishmaniasis (ATL) [12].

The Purus Region (PR) is a large area covering the states of Amazonas and Acre and has faced, throughout its history, several socioenvironmental impacts that have influenced its demographic composition, the ways of life of the human populations and the interaction with other living beings which make up the natural and anthropized environment. Latex (*Hevea brasiliensis*) extraction, which shaped economic production in the first decades of the twentieth century together with the harvesting of Brazil nuts (*Bertholettia excelsa)*, coexisted with the household-family forms of subsistence based on small-scale extractivism practiced by the innumerable indigenous and *mestizo* families who settled along the course of the Purus River, mainly in the middle third of this river [14]. For these families, the basis of economic activity continues to be the extraction of vegetable oils *Copaifera langsdorffii* and *Carapa guianensis* (copaíba and andiroba) and, above all, the seasonal harvesting of Brazil nuts from *Bertholettia excelsa*. Such a work regime implies a long stay in the jungle environment and exposure to insects, vectors and other animals of the forest [15].

Since we have little information about leishmaniasis in the Purus region (PR) [9], the aim of this study is to analyze factors related to the occurrence of ATL in the aforementioned region, based on the distribution of cases reported by its municipalities, considering a period of 13 years: 2001 to 2013. We sought to correlate the cases with the ways of living and subsistence farming in this region. Knowledge of these aspects of this endemic disease will certainly contribute to the improvement of control strategies in the region.

## Materials and methods

**Fig 1:**
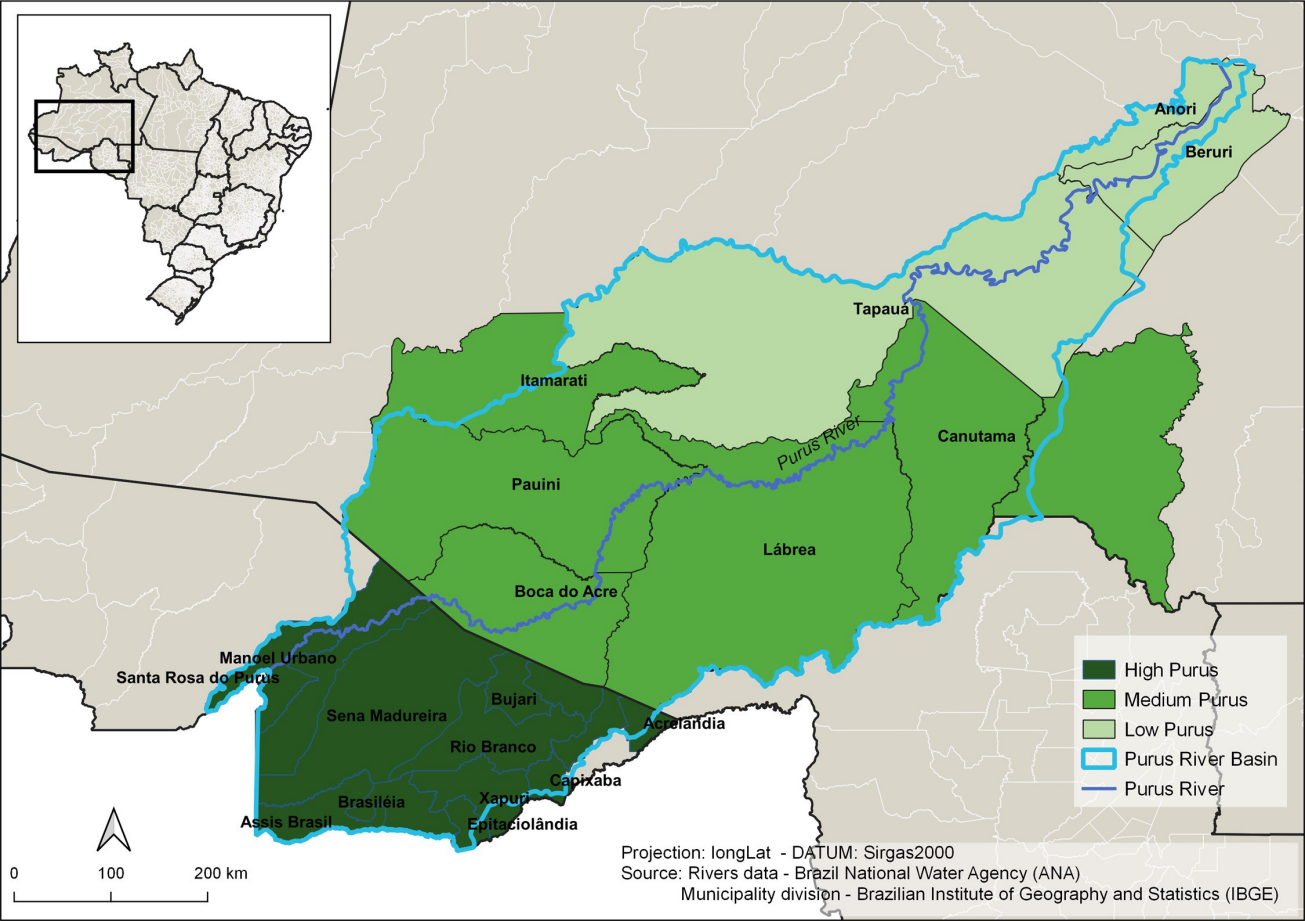
Descriptive map of the Purus region.

This is a descriptive and exploratory study of ATL cases from the PR, which is composed of three sub-regions: Upper, Middle and Lower Purus, which comprise of eight municipalities within the state of Amazonas: Anori, Beruri, Boca do Acre, Canutama, Itamarati, Lábrea, Pauiní and Tapauá and thirteen municipalities in the state of Acre: Acrelândia, Assis Brasil, Brasilia, Bujari, Capixaba, Epitaciolândia, Manoel Urbano, Porto Acre, Rio Branco, Santa Rosa do Purus, Sena Madureira, Senator Guiomard and Xapuri (Fig 1).

The monthly number of ATL cases from the PR was compiled via secondary data obtained from the SINAN database (http://sinan.saude.gov.br/sinan/login/login.jsf) provided by the Amazonas State Health Surveillance Foundation (FVS-AM) and the ATL case notification database of the State Health Department of Acre, for the period of 2001 to 2013.

For the general ATL characterization in PR, the following variables were considered: sex, age, clinical form and occupation, the latter being adapted and organized into groups, according to the Brazilian Institute of Geography and Statistics (IBGE) Classification of Occupations for Household Surveys [16]. However, for adjustment purposes, individuals were considered as extractivists if they lived in rural areas which are already recognized as areas of transmission of leishmaniasis, and for which there was no record of the field of activity attributed to that individual.

The cumulative data of ATL cases was compared with the rainfall indexes obtained from the Lábrea municipal weather station for the stated period [17]. The association between LTA cases and each of the three prioritized variables (Human Development Index-HDI, Precipitation and Temperature) was calculated based on the incidence of cases in the municipalities.

For analysis of the association between incidence and explanatory variables, univariable linear regressions were carried out for average accumulated rainfall (mm^3^), average temperature (°C) and Municipal Human Development Index (MHDI), estimated to the municipal level in Brazil [18] and showing the degree of economic development and the quality of life offered to the population on a scale of 0 (no human development) to 1 (total human development). In the calculation of the HDI, the following factors are computed: education (average years of study), longevity (population life expectancy) and per capita Gross Domestic Product.

Municipalities were considered as a unit of analysis. Since the data does not present normal distribution, log transformation was applied in order to normalize them. For the statistical analyses R software version 3.4.0 was used.

## Ethical aspects

The National Ethics Research Council and Research Ethics Committee of the Tropical Medicine Foundation Heitor Vieira Dourado, Manaus Amazonas, authorized this work to be based on secondary data, under the Ethics Licence number: 57930.216.5.0000.0005, which dispensed with the requirement for informed consent. All the data used in the present study was anonymized and only variables such as number of cases, origin by municipality, age group, sex and clinical form were used. The records containing such data and the consent were requested from the Health Surveillance Department (DVISA) of the respective States.

## Results

### Occurrence of ATL by region and period

For the period studied, 13,971 cases of ATL were registered and distributed among the three sub-regions of the Purus: Upper Purus: 12,611 (90.27%) where the incidence rates are higher, Middle Purus: 1,225 (8.77%) and Lower Purus: 135 (0.96%). Among the 21 different municipalities of PR, attention is drawn to Rio Branco, with 31.7% of all cases, followed by Xapuri (12.6%) and Sena Madureira (12.5%). The predominant clinical form was cutaneous (78.8%), however, the general percentage of mucosal forms (20.8%) is remarkable (Table 1).

**Table 1:** Distribution of cases of ATL in PR according to the municipalities of notification and clinical forms of each sub-region.

	Clinical form									
	Cutaneous	%	Mucosal	%	NR	%	Total	%		
**Upper Purus**	**9,891**	***78*.*4***	**2,689**	***21*.*3***	**31**	***0*.*3***	**12,611**	***90*.*3***	**Average population**	**Incidence X 10.000 hab.**
Acrelândia	288	*2*.*3*	17	*0*.*1*	0	*0*,.*0*	305	*2*.*2*	12538	243.3
Assis Brasil	572	*4*.*5*	208	*1*.*6*	0	*0*.*0*	780	*5*.*6*	6072	1284.6
Brasiléia	900	*7*.*1*	150	*1*.*2*	4	*0*.*0*	1,054	*7*.*5*	21398	492.6
Bujari	209	*1*.*7*	73	*0*.*6*	1	*0*.*0*	283	*2*.*0*	8471	334.1
Capixaba	267	*2*.*1*	99	*0*.*8*	0	*0*.*0*	366	*2*.*6*	8798	416.0
Epitaciolândia	629	*5*.*0*	124	*1*.*0*	1	*0*.*0*	754	*5*.*4*	15100	499.3
Manoel Urbano	238	*1*.*9*	145	*1*.*1*	1	*0*.*0*	384	*2*.*7*	7981	481.1
Plácido de Castro	6	*0*.*0*	1	*0*.*0*	0	*0*.*0*	7	*0*.*1*	17209	4.1
Porto Acre	319	*2*.*5*	79	*0*.*6*	4	*0*.*0*	402	*2*.*9*	14880	270.2
Rio Branco	3,624	*28*.*7*	793	*6*.*3*	7	*0*.*1*	4,424	*31*.*7*	336038	131.7
Santa Rosa do Purus	124	*1*.*0*	36	*0*.*3*	0	*0*.*0*	160	*1*.*1*	4691	341.1
Sena Madureira	1,241	*9*.*8*	492	*3*.*9*	8	*0*.*1*	1,741	*12*.*5*	38029	457.8
Senador Guiomard	156	*1*.*2*	28	*0*,.*2*	0	*0*.*0*	184	*1*.*3*	20179	91.2
Xapuri	1,318	*10*.*5*	444	*3*.*5*	5	*0*.*0*	1,767	*12*.*6*	16091	1098.1
**Middle Purus**	1,008	***82*.*3***	209	***17*.*1***	8	***0*.*7***	1,225	***8*.*8***	**37677**	**438.9**
Boca do Acre	433	*35*.*3*	127	*10*.*4*	6	*0*.*5*	566	*4*.*1*	30632	184.8
Canutama	39	*3*.*2*	4	*0*.*3*	0	*0*.*0*	43	*0*.*3*	12738	33.8
Itamarati	22	*1*.*8*	5	*0*.*4*	0	*0*.*0*	27	*0*.*2*	8038	33.6
Lábrea	424	*34*.*6*	47	*3*.*8*	2	*0*.*2*	473	*3*.*4*	37701	124.5
Pauiní	90	*7*.*3*	26	*2*.*1*	0	*0*.*0*	116	*0*.*8*	18166	63.9
**Lower Purus**	116	***85*.*9***	14	***10*.*4***	5	***0*.*0***	135	***1*.*0***	**107275**	**88.3**
Anori	5	*3*.*7*	0	*0*.*0*	0	*0*.*0*	5	*0*.*0*	16317	3.1
Beruri	6	*4*.*4*	2	*1*.*5*	0	*0*.*0*	8	*0*.*1*	15486	5.2
Tapauá	105	*77*.*8*	12	*8*.*9*	5	*3*.*7*	122	*0*.*9*	19077	64.0
**Total**	**11,015**	***78*.*83***	**2,912**	***20*.*84***	**44**	***0*.*31***	**13,971**	***100***	**50880**	**72.2**

In the annual distribution, in 2004, there was a larger case register, with 1,524 (10.9%) cases recorded and the lowest number of cases per year was in 2001, with 745 (5.3%) (Table 2).

**Table 2:** Annual distribution of cases ATL in the PR.

Year	Number of Cases			
	Upper Purus	Middle Purus	Lower Purus	Total
**2001**	683	40	22	745
**2002**	1,069	91	21	1,181
**2003**	1,337	56	13	1,406
**2004**	1,429	81	14	1,524
**2005**	1,286	93	11	1,39
**2006**	1,059	106	6	1,171
**2007**	767	73	0	840
**2008**	843	113	8	964
**2009**	793	67	6	866
**2010**	919	127	5	1,051
**2011**	720	98	9	827
**2012**	920	148	18	1,086
**2013**	786	132	2	920
**No. of Cases**	12,611	1,225	135	13,971

### Climate variables

**Fig 2:**
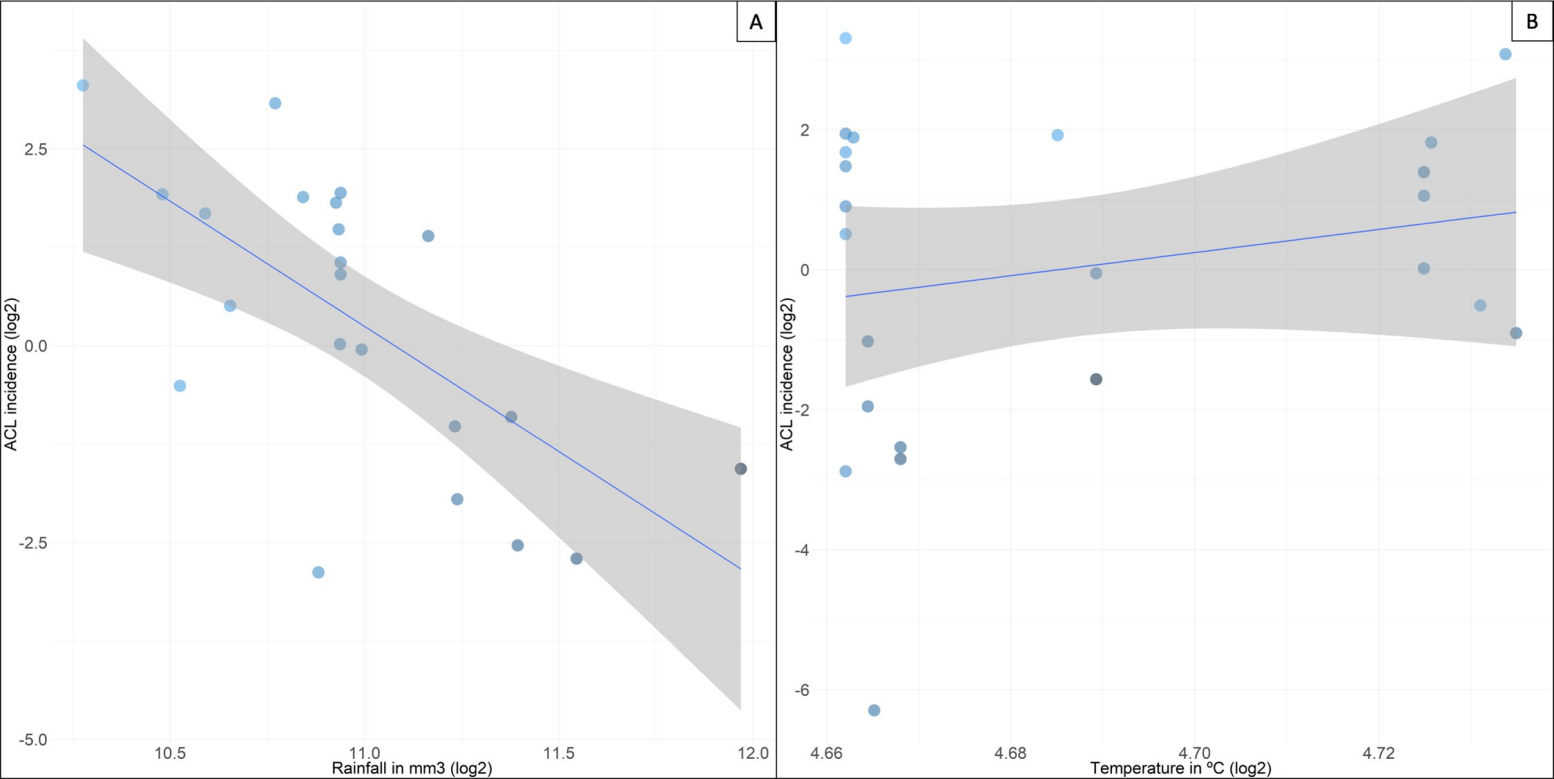
Linear regression for ATL incidence. (A) ATL Incidence vs. Rainfall. (B) ATL Incidence vs. Temperature.

A statistically significant negative association (Coef = -3.182, p-value < 0.01) was demonstrated between cutaneous leishmaniasis (CL) and rainfall between Purus municipalities. However, for temperature (Coef = 9.458, p-value > 0.05) no significant associations were found in the Purus region (Figs 2 and S2).

#### Age groups

**Fig 3:**
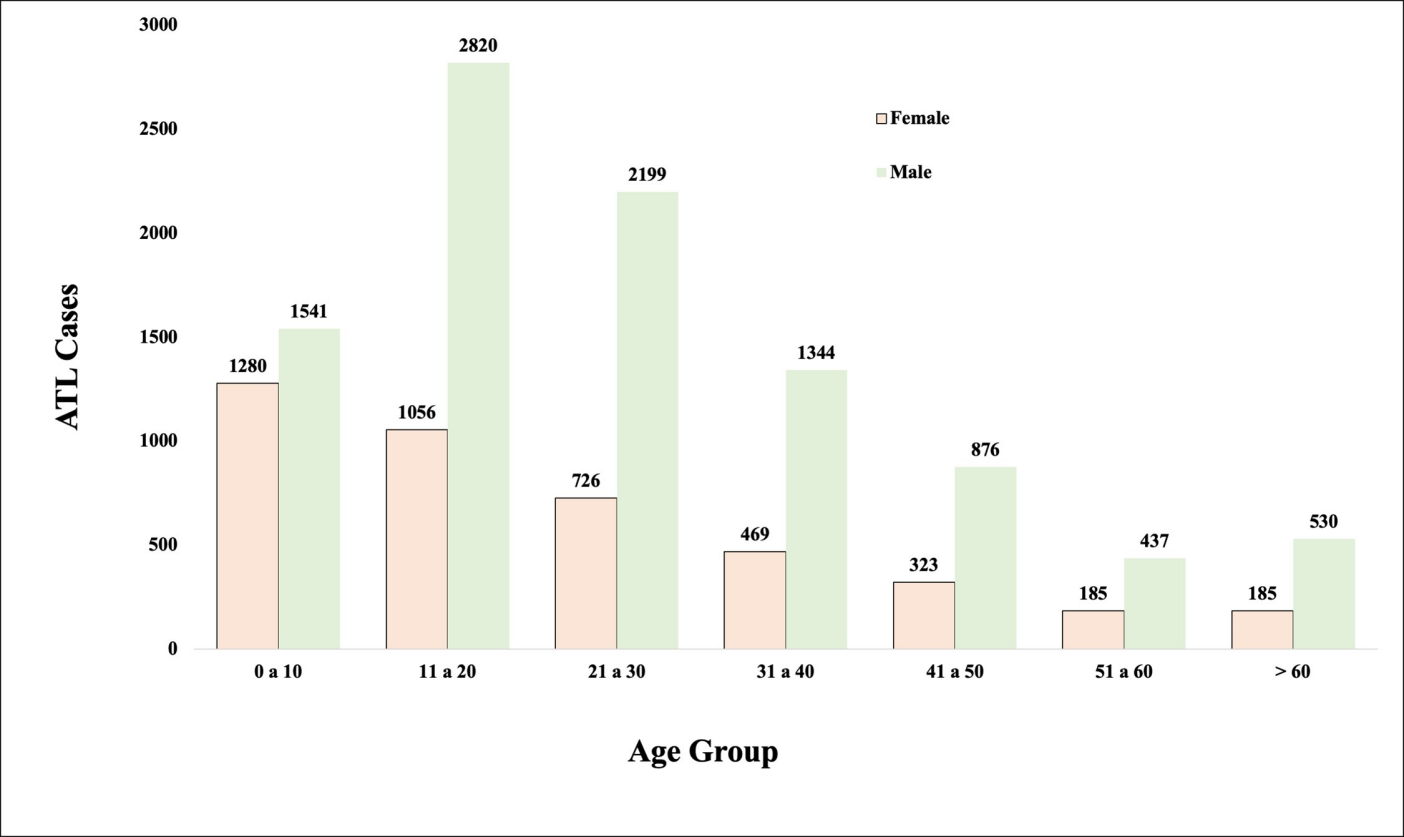
Distribution of cases of ATL in PR by age group.

In the age group analysis, there was a greater number of notifications in the age group of 11 to 50 years of age, which represented 70.2% of the cases. In the distribution by gender, there was a predominance of males, with 9747 cases (69.8%) (Fig 3).

#### Occupation

**Fig 4:**
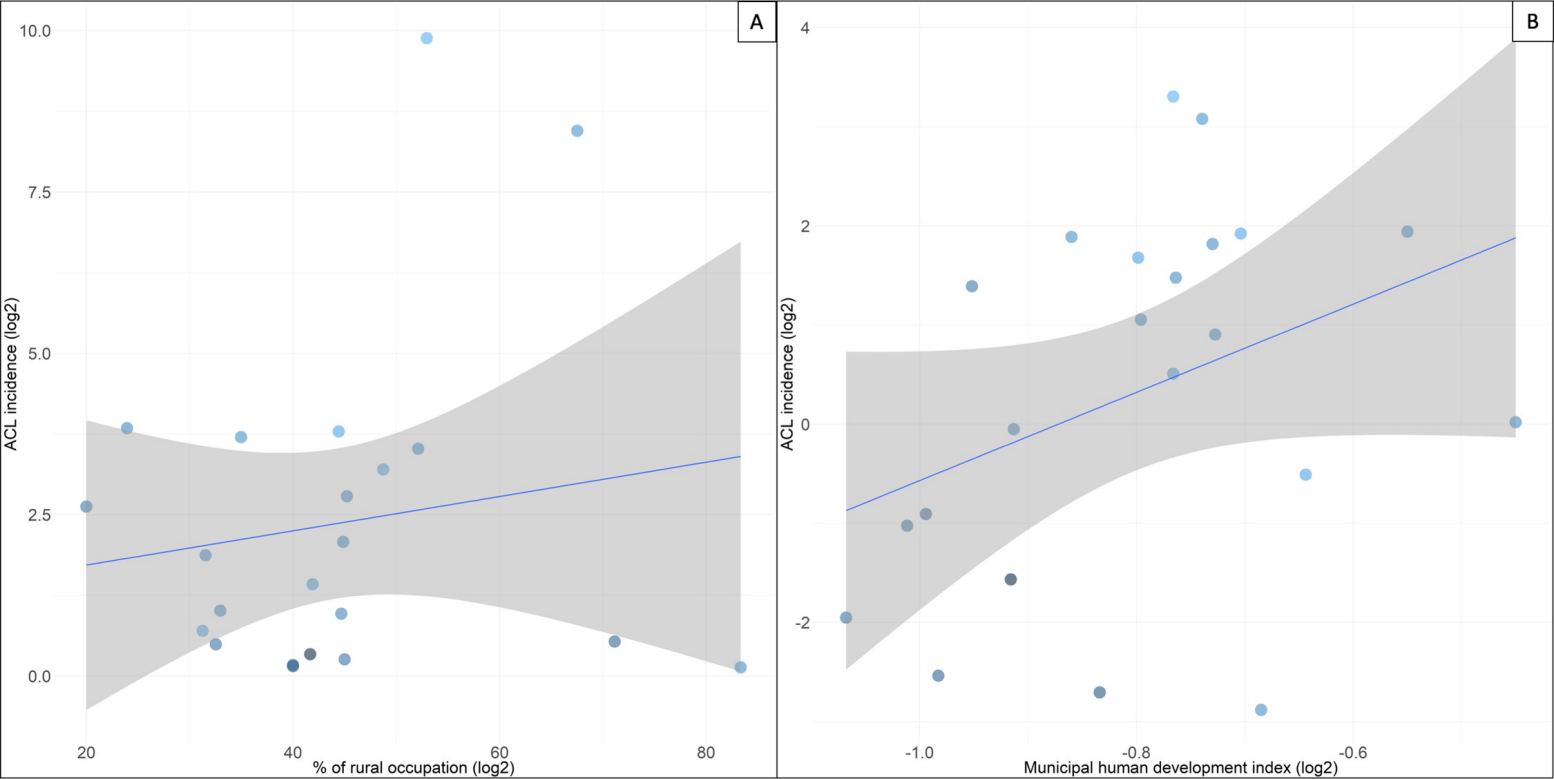
Linear regression for ATL incidence. (A) ATL incidence vs. rural occupation. (B) ATL incidence vs. MHDI.

In the distribution of notifications according to the occupation, the group of agricultural, hunting, and fishing workers (extractivism) stands out with 34% of cases registered (Table 3), withno significant associations (Coef = 0,831 p-value>0.05). In the MHDI analysis (Coef = 4.454, p-value <0.10), low significant associations were found in the Purus region (Figs 4 and S2).

**Table 3:** ATL cases by occupation groups—2001 to 2013.

Groups*	Cases	%
Agricultural, forestry, hunting and fishing workers	4744	34.0
No information in database	3358	24.0
Teenagers	2662	19.1
Workers in the production of industrial goods and services	1596	11.4
Student	556	4.0
Household and general trade workers	282	2.0
Administrative service workers	266	1.9
Professionals of science and the arts	194	1.4
Repair and maintenance workers	169	1.2
Members of the armed forces, police and military firefighters	71	0.5
Other ill-defined occupations	61	0.4
Middle level technicians	12	0.1
**Total**	**13971**	**100**

(*) according to IBGE classification

## Discussion

### Occurrence of ATL by region and period

This study describes the occurrence of leishmaniasis in the Amazon Purus region, where the process of forest destruction, conflicts and violence against its inhabitants is the spearhead of a model of implantation of agricultural and livestock monocultures in the Amazon; a developmental scheme that is present in the sub-region of the Upper Purus, in the municipalities that make up the state of Acre, and now also advances in the south of the Amazon, thus putting pressure on the protected areas and the territory of the people and communities, and causing damage and compromising their natural resources [14].

The economy of the state of Acre is based primarily on the extraction of latex and Brazil nuts, and also on livestock. Acre is the largest producer of latex in the country, with rubber trees being found mainly in the Purus, Juruá and Madeira river basins.

The harvesting of the Brazil nut is an activity also carried out by rubber tappers, and this harvesting occurs during the rainy season, adding important value to the agroextractivist production in general, and thus consolidating the predominance of the so-called "temporary agroextractivist" (which includes not only the harvesting of Brazil nuts, but other agroextractivist activities such as planting of vegetables during the dry season of the rivers, hunting, and extraction of copaiba, andiroba types of vegetable oils—all activities that include entry into the forest and are carried out by the communities that live there. This culture of agroextractivist production, previously considered temporary, adds around 40 to 47% of total agroextractivist production in general (hence its importance) in this context and in the economic context of the Purus region, all of which refers to exposure and consequent risk to ATL [19].

In large cities, and especially on the periphery of these, the degree of exposure of affected individuals is directly related to processes of disordered occupation or to the so-called invasions. This is a different scenario from that described here, especially for the Middle and Lower sub-regions of Purus, but not for the Upper Purus sub-region, because the reality of Rio Branco and its surroundings is more consistent with that of Manaus and other cities where ATL generally occurs in places of recent population settlements and is related to deforestation, or in the vicinities along the roads, in populations close to areas of primary forest, and due to the construction of disorganized housing and without any planning by city departments. Individuals are exposed to the vectors, and later become endemic to sporadic cases [3,7,11,20–22]. From 2001 to 2007, the mean annual number of cases in the Lower and Middle Purus sub-regions was 90 cases, in the period from 2008 to 2013 this average rose to 122 cases. In the Upper Purus sub-region we observed the inverse, with a mean of 1090 in this same first period, to 890 in the second.

The growth of the city of Rio Branco, the capital of the state of Acre, has been identified as the main factor responsible for the greater number of cases registered in that sub-region, as was the case with Manaus [7,22]. This is reflected in the comparison of activities among the Purus Sub-regions [23], where there is a difference between the activities of the patients: the Upper Purus presents a greater number of cases among the factory workers producing industrial goods and providing services in comparison to the cases of the same category in the other sub-regions (Table 3).

In the temporal distribution of ATL cases, an average of 1,074 cases per year is observed, but this reflects a general fact for the entire Northern Region, taking into account the hyper-endemic years of 2003 and 2004 (although this is also consistent with the increase in deforestation in the municipality of Lábrea, which presented an average for deforestation which was 236% higher for the three-year deforestation period 2004/2006 than the 2001/2003 period).

The increase in the number of ATL cases during the period also suggests that the expansion of access to health services, the quality of the diagnosis and the notification system in the PR improved [22,24]. These actions are very important for surveillance of the mucosal form of the disease, which accounts for 20.8% of PR case reports; a very high rate, since the development of the mucosal form may arise when the cutaneous form is not adequately treated, usually due to the patient’s lack of knowledge and difficulty of access to the health service for diagnosis and treatment.

### Climatic variables

Chaves and Pascual [25] showed that in Costa Rica (Central America) Cutaneous Leishmaniasis dynamics are strongly associated with climatic variables. In the Amazon region, especially in the PR, throughout the year there are no large variations in temperature and this variant does not have a positive or negative influence on the occurrence of cases. However, when rainfall is associated with the incidence of ATL cases, data analysis showed a negative correlation, different from what is observed in the region of Manaus, where the ATL cases predominate at the beginning and end of each year, corresponding to the period of greater rainfall in the region (28). Phenomena such as "El Niño” may interfere in the cycle of transmission of vector diseases by increasing or decreasing levels of rainfall. These changes in rain fall cause a change in the density of sand flies, as observed in other studies [26,27]. In Panama, a positive association between precipitation and ATL cases was also observed [27].

In the Purus Region, ATL transmission intensifies during the dry season when the extraction of the latex is carried out, however, it also occurs during the rainy season when the harvesting of the Brazil nuts is done. The frequent trips to the forest, as well as the stay in the forest, both necessary in order to carry out these activities, represent exposure to the vectors and the consequent risk of acquiring the disease. The notifications tend to be carried out at a later period, due to the slow evolution of the clinical illness and the prolonged incubation period, associated with difficulties of access to the health services for diagnosis.

It can also be noted that the cycle of agroextractivist activities of the riverine populations determines the continuation of the endemic throughout the year, either due to deforestation for the planting of crops, or due the constant decamping for the subsistence activities carried out by this population [28–30]. In terms of the extractive sector, we observed that the highest incidence rates of ATL correlate with the municipalities that present the highest production of latex and largest harvests of Brazil nuts.

Although the traditional extractive form of exploration does not fit into the modern systems of globalization or is affected by the advancement of technology and the expansion of more profitable products, with an increase in the need for wood, the current state policy has made the state of Acre turn even more towards the exploitation of the Brazilian Amazon, and even under the discourse of sustainable management, the positive correlations between the volume of deforestation and logging shows the possibility of this management not being as ecologically positive as that of official propaganda [19].

### Age group

The age group most affected (11 to 50 years old), at a rate of 70.2%, is the age range with the highest labor intensity and, therefore, is the most exposed [20]. However, the cases in the age group from zero to 10 years (20.2%), and cases in the elderly (5.1% in the age group over 61 years), suggest the occurrence of transmission in the peri and / or household environment [11,21,22]. This may be related to the construction of residences in the vicinity of the forest areas. The presence of domestic animals in the peridomicile, such as dogs, chickens, pigs, cows, etc., also serves as an attraction to hematophagous insect vectors and consequently to human infection in these places [5,28].

### Occupation

The participation of the whole family, including women and children, is common, at least in some stages of the production process, such as the harvesting of wood for domestic use, for sale and for making charcoal, the search for and harvesting of food in the jungle and in the waterways, especially Brazil nut collection, and assisting of parents in agricultural activities, hunting and fishing are also important factors in the transmission of ATL [21,29–31]. In short, the presence of children and young people in productive activities that involve deep and recurrent interaction with the jungle environment is common, which, in this respect, differs from adolescents in cities [15].

The predominance of ATL in 9,394 individuals or 43.7% of the cases that have their occupational activities related to vegetal extraction, deforestation, road construction, military training, oil drilling expeditions, corroborate with other studies that suggest a greater risk for the acquisition of ATL due to these labor activities in the forest [31–34]. Another social factor predisposing people to ATL exposure is the fact that, in the Purus context, adolescents work as adults, thus sharing the same risk factors as their older relatives, which is why this is the age group responsible for 19.1% of the cases (Table 3).

In recent years, the flow of *mestizo* and indigenous families who have moved towards the cities has increased in a very expressive way, thus modifying the extractive pattern for a model landowner. Such dynamics imply not only a change of scenery, but also a change of social flows [35]. Even indigenous communities are heavily influenced by these changes, and in recent years there has been an intensification of the urbanization of previously settled indigenous populations.

Perhaps this difference is associated with the economic activity of the rural population in PR, which includes an entrance and a stay in forest regions, such as the already mentioned Brazil nut harvesting, and the tapping of latex, the mining, and the extraction of wood during the dry period. Most of the time the high rainfall prevents the exercise of those activities that are the main exposure factors for ATL.

This traditional way of life, which remains alive and functioning, shaped by the fusion between Northeastern migrants and indigenous elements since the first decades of the twentieth century, is periodically shaken by the economic movement from the non-indigenous world. In the 1970s, the construction of highways returned to mess up the local social structure, also exerting a strong influence on the occurrence of diseases and other social problems [36]. The 1990s redefined the political and territorial profile of the Purus Region (PR), with the affluence of agricultural and hydroelectric enterprises which, guided by business strategies, exploited the region in an attempt to produce commodities for exportation (with emphasis on livestock production, grain production, timber sales and hydroelectric power generation, and with a considerable environmental impact generated by deforestation and severely threatening the lives of the traditional populations settled there) [36]. Consequently, municipalities such as Lábrea and Canutama, both located in the state of Amazonas, are highlighted in the ranking of municipalities with high rates of deforestation in the Amazon. Between 2003 and 2004, there was a large increase in deforested areas in Lábrea (from 175.07 km^2^ in 2003 to 328.97 km^2^ in 2004), followed by Canutama, also with high deforestation, a situation that included the region of Purus as a critical area in the arc of the development of the Amazon [35].

In the south of the municipality of Lábrea, there are numerous areas of cattle ranching, with consequent deforestation in the stretch between Humaitá and Lábrea, both along the BR 230 and in the lowland of the Purus river. In these places, the presence of large ranchers is observed, driving out extractive communities and family farmers. In Canutama, traditional crops give way to the mechanized culture of soybeans, mainly in the south of this municipality [36].

The existence of hardwood, especially in Lábrea, also emerges as a factor to accelerate deforestation. Between 2003 and 2004, this municipality increased its deforested area by 87%, due to the presence of cedar and mahogany in its forests, especially around BR 364 highway (Porto Velho / Rio Branco) and BR 230 –Transamazonian highway. In the south of the municipality of Lábrea, there are 43 sawmills located in municipalities on the border with Rondônia (RO), spread along BR 364. According to Greenpeace data, in 1999, Amazonian wood jumped from 14% to 85% of the national production [36].

The negative impact on the traditional ways of life of the PR populations has intensified, producing, in return, the outbreak of movements of territorialization associated with the presence of traditional extractive people and practices, supported by environmental NGOs. Although relevant to the preservation of the livelihoods of local populations, such initiatives have not been sufficient to reverse the predatory process, often leading to forced mobilization of populations that have long been settled and stabilized in local ecosystems.

Thus, the context described above shows that PR has, over the decades, maintained favorable social and environmental conditions to the production of endemics such as ATL, strongly related to the productive cycles described, to the uncontrolled processes of deforestation and to the mobilization of population masses previously settled more permanently in their traditional territories [14].

Although it has been demonstrated elsewhere, associations of ATL incidence with temperature and the human development index were not significant in the Purus region during the period of the study. This data indicates that there is another factor associated with the high number of registered cases and we can say that PR has maintained the same high level of endemicity, especially related to cases of LM.

ATL mainly affects populations which have low income and low grade schooling, and mainly live in the Amazon. The vast majority of individuals who acquire the disease were developing work within the forest, mainly extractivism, which constitutes a fundamental strategy for survival for the Amazonian man [12]. From 1985 to 1997, the occurrence of ATL cases in Manaus reached 56% of the total occurred in Amazonas, and the disease occurred mainly in epidemic outbreaks [3,24].

## Conclusion

In conclusion, we can attribute the occurrence and maintenance of ATL in PR to two different well-known elements [36]: on the one hand, the "development companies", represented by the advancement of the agricultural and cattle frontier in waves originating from the states of Rondônia, Amazonas and Acre, which are stimulated by public policies that encourage urban development, land invasion, road making, construction of hydroelectric plants and waterways. On the other hand, we can see the social-environmental vector, manifested in the maintenance of traditional actors in this scenario, who use, above all, extractivism as a source of income and subsistence. This extractivism is mainly marked by the tapping of latex and Brazil nut harvesting, but also, by a background that includes the extraction of lumber, changes in lifestyle, difficulties of access to health services and environmental impacts.

## Supporting information

S1 Table Gross data of study variables. Worksheet with raw data of variables: date of notification, municipalities, age, sex and occupation.
(XLSX)

S1 Fig Linear regression for ATL incidence. Worksheet with raw data of variables: cases per year by municipality, MHDI, population, rainfall and temperature.
(XLSX)

S2 Fig Linear regression for ATL incidence. Statistical analysis using software R version 3.4.0.
(XLSX)
